# Therapeutic effects of human umbilical cord blood-derived mesenchymal stem cells after intrathecal administration by lumbar puncture in a rat model of cerebral ischemia

**DOI:** 10.1186/scrt79

**Published:** 2011-09-22

**Authors:** Jung Yeon Lim, Chang Hyun Jeong, Jin Ae Jun, Seong Muk Kim, Chung Heon Ryu, Yun Hou, Wonil Oh, Jong Wook Chang, Sin-Soo Jeun

**Affiliations:** 1 Department of Biomedical Science, College of Medicine, The Catholic University of Korea, Seoul, 137-701, Korea; 2 Department of Neurosurgery, Seoul St. Mary's Hospital, The Catholic University of Korea, Seoul, 137-701, Korea; 3 Medipost Biomedical Research Institute, MEDIPOST Co., Ltd., Seoul, 137-073, Korea

## Abstract

**Introduction:**

Stem cell transplantation is a promising therapeutic strategy for the treatment of stroke. Mesenchymal stem cells (MSCs) are a potential cell source for clinical application because they can be easily obtained and cultivated with a high proliferative capacity. The safety and efficacy of cell therapy depends on the mode of cell administration. To determine the therapeutic potential of intrathecal administration of MSCs by lumbar puncture (LP), we administrated human umbilical cord blood-derived MSCs (hUCB-MSCs) intrathecally into the lumbar spinal cord or intravenously into the tail vein in a rat model of stroke, and then investigated whether hUCB-MSCs could enter the brain, survive, and improve post-stroke neurological functional recovery.

**Methods:**

hUCB-MSCs (1.0 × 10^6^) were administrated three days after stroke induced by occlusion of the middle cerebral artery. The presence of hUCB-MSCs and their survival and differentiation in the brain tissue of the rats was examined by immunohistochemistry. Recovery of coordination of movement after administration of hUCB-MSCs was examined using a Rotarod test and adhesive-removal test on the 7^th^, 14^th^, 21^st^, and 28^th ^days after ischemia. The volume of ischemic lesions seven days after the experimental procedure was evaluated using 2-3-5-triphenyltetrazolium (TTC) staining.

**Results:**

Rats receiving hUCB-MSCs intrathecally by LP had a significantly higher number of migrated cells within the ischemic area when compared with animals receiving cells intravenously. In addition, many of the cells administered intrathecally survived and a subset of them expressed mature neural-lineage markers, including the mature neuron marker NeuN and glial fibrillary acidic protein, typical of astrocytes. Animals that received hUCB-MSCs had significantly improved motor function and reduced ischemic damage when compared with untreated control animals. Regardless of the administration route, the group treated with 1 × 10^6 ^hUCB-MSCs showed better neurological recovery, without significant differences between the two treatment groups. Importantly, intrathecal administration of 5 × 10^5 ^hUCB-MSCs significantly reduced ischemic damage, but not in the intravenously treated group. Furthermore, the cells administered intrathecally survived and migrated into the ischemic area more extensively, and differentiated significantly into neurons and astrocytes.

**Conclusions:**

Together, these results indicate that intrathecal administration of MSCs by LP may be useful and feasible for MSCs treatment of brain injuries, such as stroke, or neurodegenerative disorders.

## Introduction

Major human brain and spinal cord injury remain serious problems that currently have no effective treatment. Stem cells have the potential to induce neurorestorative processes, including neurogenesis, angiogenesis, and synaptic plasticity that are essential for facilitating recovery of neurological function [1]. Therefore, transplantation of stem cells is a promising therapeutic strategy for the treatment of many neurological disorders.

Mesenchymal stem cells (MSCs) are highly attractive candidates for the application of tissue engineering to regenerate damaged tissue, because they self-renew with a high proliferative capacity and have the ability to differentiate into multiple lineages [2-7] and migrate into injured organs [8,9]. Moreover, MSCs are not immunogenic, and so they do not elicit the proliferative response of allogeneic lymphocytes *in vitro *[10].

Different routes of MSC administration have been used to treat damaged ischemic brain tissue. In many studies to date, MSCs have been injected directly into pathological regions [11-13]. When transplanted into the striatum of rats with ischemia from middle cerebral artery occlusion (MCAO), MSCs are well engrafted and migrate to the ischemic cortex. In addition, MSCs differentiate into cells that stain positive for neural markers and significantly improve motor recovery [8,14]. However, this technique raises the possibility of additional trauma resulting from transplantation surgery, leading to a reduced survival of grafted cells. Moreover, this surgical procedure is often impractical for patients whose condition is clinically severe [15]. Furthermore, direct parenchymal cell transplantation does not allow delivery of multiple doses of therapeutic cells.

Intravenous infusion of cells is comparatively the least invasive approach and the intravenous route is well tolerated [16]. Because there is long-term functional improvement following intravenous MSC injection in animal models of stroke, MSCs are now widely administered via this route [8,17]. The intravenous infusion of MSCs might be a feasible and safe mode for MSC treatment of stroke patients. However, many cells are distributed widely throughout the body, such as in the liver, the spleen, and the kidneys [18,19]. These concerns were addressed in a study that involved intravenous cell transplantation [20]. The authors injected MSCs intravenously following experimental traumatic brain injury, but could only demonstrate the presence of a few cells at the injury site.

The development of a safe and effective strategy for cell transplantation has been a major clinical challenge in cell therapy. Therefore, we have been investigating alternative, effective, and clinically applicable strategies for MSC delivery in a rat model of cerebral infarction. Some studies have shown that intrathecal delivery by lumbar puncture (LP) is an extremely attractive means of delivery of chemicals into the cerebrospinal fluid (CSF) and that this route is well tolerated [16]. However, little data are available regarding the delivery of cells intrathecally by LP.

To determine the possibility of delivering human umbilical cord blood-derived MSCs (hUCB-MSCs) intrathecally by LP in an MCAO model of stroke in the rat, the present study investigated the therapeutic effects and grafts of intrathecally delivered hUCB-MSCs when compared with intravenously delivered hUCB-MSCs.

## Materials and methods

### Culture of hUCB-MSCs

Human UCB samples were collected from the umbilical vein of deliveries with informed maternal consent. The 16-gauge needle of a UCB collection bag containing 44.8 ml of CPDA-1 anticoagulant (Greencross, Yongin, Korea) was inserted into the umbilical vein and UCBs were collected by gravity. Isolation and expansion of UCB-MSCs was conducted as previously reported [21]. In brief, mononuclear cells were isolated by centrifugation in a Ficoll-Hypaque gradient (density 1.077 g/cm3, Sigma, St Louis, MO, USA). The separated mononuclear cells were washed, suspended in a-minimum essential medium (a-MEM; Gibco BRL, Carlsbad, CA, USA), supplemented with 10% fetal bovine serum (FBS; HyClone, Logan, UT, USA), and seeded at a concentration of 5 × 10^6 ^cells/cm^2^. Cultures were maintained at 37°C in a humidified atmosphere containing 5% CO_2 _with a change of culture medium twice a week. UCB-derived mononuclear cells were set in culture, and the onset of fibroblast-like adherent cells was observed. One to three weeks later, when the monolayer of MSC colonies reached 80% confluence, cells were trypsinized (0.25% trypsin, HyClone), washed, resuspended in culture medium (a-MEM supplemented with 10% FBS) and subcultured at a concentration of 5 × 10^4 ^cells/cm^2^. MSCs of each UCB harvest were expanded *ex vivo *by successive subcultivation under the same condition. The fifth to eighth passage cells of UCB harvests with more than 1,000-fold expanding capacity were used for the experimental work. Ethical approval for the use of hUCB-MSCs was obtained from the Institutional Review Board of Catholic University Medical Center.

### Ischemic animal model and experimental groups

All animal protocols were approved by the Institutional Animal Care and Use Committee of the Catholic University Medical School. Anesthesia of adult male Sprague Dawley rats weighing 250 to 270 g was induced with 5% isoflurane in 70% nitrous oxide and 30% oxygen using an induction chamber, and anesthesia was maintained by supplying 1.5% isoflurane using a face mask. Rectal temperature was maintained at 37°C throughout the surgical procedure, as monitored by an electronic temperature controller linked to a heating pad (FHC, Bowdoinham, ME, USA). Transient MCAO was induced as previously described by [22], with a slight modification. Briefly, the right common carotid artery (CCA), external carotid artery (ECA), and internal carotid artery (ICA) were exposed through a ventral midline incision. A 4-0 nylon monofilament suture with a rounded tip was introduced into the CCA lumen and gently advanced into the ICA until it blocked the bifurcating origin of the MCA. Two hours after occlusion, animals were reanesthetized and reperfused by withdrawing the suture until its tip cleared the lumen of the CCA.

All experiments were randomized. Seventy-four rats that underwent a transient MCAO were directly used to obtain the final data shown in this study: intrathecal injection of phosphate buffered saline (PBS) into CSF by LP (*n *= 12), intravenous injection of PBS into tail vein (*n *= 12), intrathecal injection of hUCB-MSCs (*n *= 25), and intravenous injection of hUCB-MSCs (*n *= 25).

### Cell transplantation

hUCB-MSCs were injected intrathecally or intravenously at three days after MCAO. For intrathecal injection, LP was performed after establishment of isoflurane (inhalation) anesthesia. Briefly, each rat was anesthetized and placed on an operating surface that flexed the animal's back. A small (1 cm) longitudinal incision was made over the L3 to L5 spinous processes and the skin was retracted. A human neonatal lumbar puncture needle (25 gauge; Becton Dickinson, Franklin Lakes, NJ, USA) was advanced into the spinal canal at the L3 to L4 or L4 to L5 level. Proper placement of the needle in the lumbar subdural space was indicated by three signs: loss of resistance at the time of entry (tentative sign), tail flick (more definitive sign), and presence of CSF in the needle hub (most definitive sign). Once correct needle placement was confirmed, the CSF present in the needle hub was aspirated using a micropipette, and hUCB-MSCs (1 × 10^6^) diluted in 20 μl PBS were injected into the CSF over 30 s. The skin was closed by stapling and the animal was returned to its cage. The entire procedure took three to five minutes. For intravenous injection, hUCB-MSCs (1 × 10^6^) diluted in 700 μl PBS were injected slowly for five minutes via an intravenous cannula situated in the tail vein.

### Immunohistochemistry and quantification

Rat brains were perfused with PBS followed by 4% paraformaldehyde under deep anesthesia at a specific time point after lumbar injection of hUCB-MSCs. The excised brains were post-fixed overnight and then equilibrated in PBS containing 30% sucrose for two days. Fixed brains were embedded, snap-frozen in liquid nitrogen, and stored at -70°C until use. Tissues were cryosectioned at 14 μm in the coronal plane and then stained with primary antibodies for neuronal nuclei (NeuN) (Chemicon, Temecula, CA, USA), human nuclei (Chemicon), and glial acidic fibrillar protein (GFAP; Dako, Glostrup, Denmark) at 4°C overnight. The sections were incubated with Alexa Fluor 488 or 546-conjugated anti-IgG secondary antibodies (Molecular Probes, Eugene, OR) and counterstained with 4',6-diamidino-2-phenylindole (DAPI; Sigma-Aldrich, St. Louis, MO). To detect apoptotic activity, tissues were stained using a terminal deoxyribonucleotidyl transferase-mediated dUTP nick end labeling (TUNEL) assay kit (Roche, Basel, Switzerland) developed with Cy3-conjugated streptavidin (Jackson ImmunoResearch Laboratories, Bar Harbor, ME, USA). Fluorescent images were acquired using a Zeiss LSM510 confocal microscope (Carl Zeiss, Jena, Germany).

To determine graft survival semiquantitatively, every fifth coronal section (15 μm) per animal was prepared and counting was performed on three randomly selected non-overlapping per section. For each section under analysis, the region of interest (ROI) was selected within the ischemic territory, and the measurement was made in a predefined field (300 μm × 300 μm). Total numbers of positive cells for the ischemic hemisphere were then obtained by multiplying by three. All images were made using an excitation filter under reflected light fluorescence microscopy (× 200 oil objective) and transferred to a computer equipped with MetaMorph software version 7.5 (Molecular Devices, Downingtown, PA, USA).

### Motor function evaluation

Animals were pretrained for one week prior to a motor test using a Rotarod cylinder (IITC Life Science, Woodland Hills, CA, USA). The cylinder was accelerated from 4 to 40 rpm within 5 minutes, and the cutoff time was 300 s. For adhesive removal tests, square dots of adhesive-backed paper (12∅) were used as bilateral tactile stimuli occupying the distal-radial region on the wrist of each forelimb. Animals were given three trials with a cutoff time of 180 s. The data are presented as the mean time to remove the left dot.

### Staining and quantitative analysis of infarct volume

Seven days after the administration of cells, all rats (*n *= 5 for each group) were deeply anesthetized with isoflurane. Transcardiac perfusion was performed with saline. The brain of each rat was immediately removed and sectioned into four equally spaced (2 mm) coronal blocks using a rodent brain matrix. These sections were stained with 2% 2,3,5-triphenylterazolium (TTC) with normal saline for 30 minutes at 37°C. The unstained area was considered to be the infarcted area [23]. The total infarct volume for each slice was calculated by summation of infarcted areas of all brain slices, using MetaMorph software (Molecular Devices).

### Statistical analysis

All data are expressed as mean ± standard error of the mean. The significance of differences between test conditions was assessed using Student's *t*-test. Probability values less than .05 were considered as significant. Behavior results were analyzed using repeated measures analysis of variance with independent variables of treatment groups and days of testing, followed by Tukey's *post hoc *test for multiple comparisons at each treatment group.

## Results

### Homing of transplanted hUCB-MSCs toward the ischemic brain

To examine the feasibility of intrathecal administration by LP for transplanting hUCB-MSCs in rats with cerebral ischemia, we tested whether intrathecally introduced hUCB-MSCs by LP would migrate and engraft into ischemic brain. Flow cytometric analysis of hUCB-MSCs indicated this cell surface phenotype (Additional file 1, Figure S1). A characteristic feature of MSCs was a CD45^-^, CD44^+^, C90^+^, SH2^+ ^(CD105), and SH3^+ ^(CD73) cell surface phenotype.

**Figure 1:**
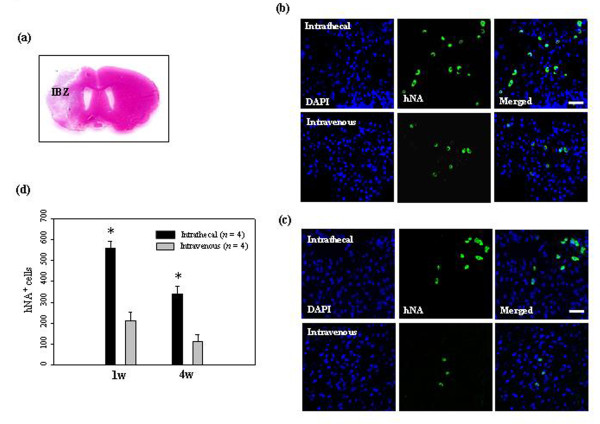
**Migration of administered hUCB-MSCs into the ischemic brain**. (**a**) Representative hematoxylin and eosin staining of coronal sections from ischemic brain. (**b**) At 7 days and (**c**) 28 days after 1 × 10^6 ^hUCB-MSC administration, hUCB-MSCs were identified by the staining with human nuclei antibody (hNA, *green*) and the numbers of hNA-positive cells in the ischemic boundary zone (IBZ) of Ipsi hemisphere are illustrated (*n *= 4 per treatment group). (**d**) Data are presented as mean numbers of hNA-positive cells ± SD. Note that the numbers of hNA-positive cells were decreased in animals after intravenous administration compared with animals after intrathecal administration. Intrathecally treated groups showed significant differences from the intravenously treated groups in the IBZ (analysis of variance; *P < 0.05). Nuclei were counterstained with DAPI (*blue*). Scale bar = 20 μm.

After inducing ischemic stroke, 1 × 10^6 ^hUCB-MSCs were injected intrathecally or intravenously and then migration of these cells was observed at 7 and 28 days in the ischemic brain (Figure 1). We identified hUCB-MSCs with anti-human nuclei antibody (hNA). A significant number of cells were found in the peri-infarct zone of the ischemic hemisphere in animals after intrathecal administration compared with animals after intravenous administration. However, administered hUCB-MSCs were not observed in the intact contralateral hemisphere after either intrathecal or intravenous delivery (data not shown).

### Survival of transplanted hUCB-MSCs *in vivo *

Insufficient graft survival and efficacy is a major obstacle in the use of MSCs for therapy. When transplanted into the striatum or tail vein after MCAO, MSCs survived and migrated to the ischemic site, but only a few of the transplanted cells survived and retained their competency [11,12,24-26].

**Figure 2:**
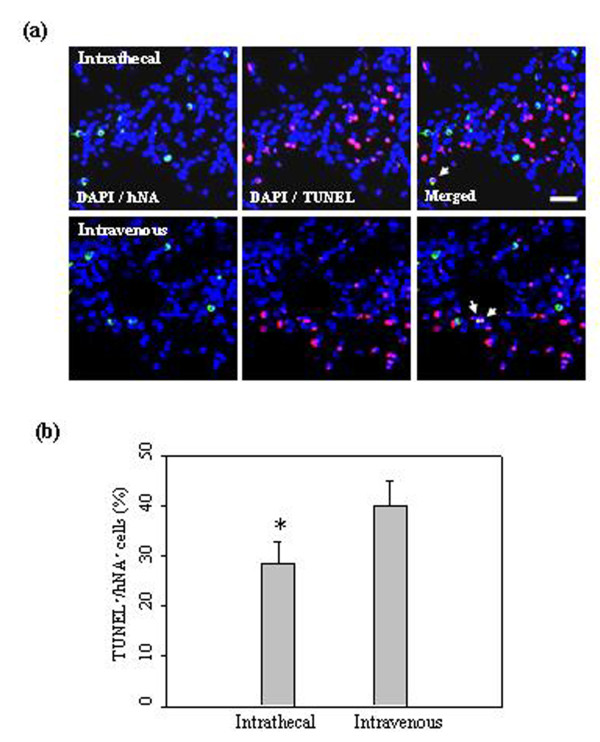
**hUCB-MSCs undergoing apoptotic cell death in the ischemic brain**. (**a**) At seven days after 1 × 10^6 ^hUCB-MSC administration, hUCB-MSCs undergoing apoptotic cell death were measured by TUNEL staining (*n *= 5 per treatment group). hUCB-MSCs were identified by the staining with human nuclei antibody (hNA, *green*). The numbers of hNA-TUNEL double-positive cells in the ipsilateral ischemic boundary zone (IBZ) are illustrated. (**b**) Quantitative analysis of hNA-TUNEL double-positive cells in the ipsilateral IBZ. Data from five animals are presented as mean values ± SD. There were significantly more hNA-TUNEL double-positive cells in animals after intravenous administration (analysis of variance; *P < 0.05). Nuclei were counterstained with DAPI (*blue*). Scale bar = 20 μm.

To assess whether hUCB-MSCs delivered intrathecally by LP would survive, a TUNEL assay was used to evaluate apoptosis of grafted cells in ischemic animals. One week after cell administration, 28% ± 4.4% of hNA-positive cells were stained for TUNEL (in the penumbra regions such as the ischemic boundary zone in animals administered hUCB-MSCs intrathecally (Figure 2a)). However, 39% ± 5.1% of hNA-positive cells were stained in animals in which they were administered intravenously (Figure 2b), which indicates that intrathecal administration of hUCB-MSCs is valuable for efficient cell delivery into the ischemic animals.

### Phenotype of transplanted hUCB-MSCs *in vivo *

**Figure 3:**
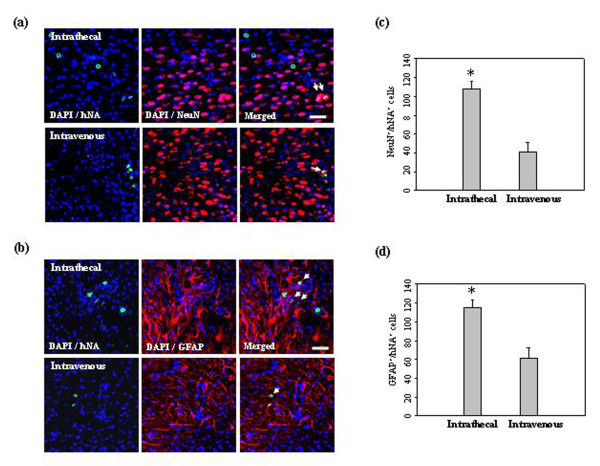
***In vivo *differentiation of hUCB-MSCs in the ischemic brain**. Confocal images of the cells at four weeks after 1 × 10^6 ^hUCB-MSC administration in the ischemic animal models. hUCB-MSCs were identified by the staining with human nuclei antibody (hNA, *green*). hUCB-MSCs survived for at least four weeks and a subset of the grafted cells expressed (**a**) NeuN and (**b**) GFAP in the ipsilateral ischemic boundary zone (IBZ). These markers were immunolabeled with red fluorescence. Quantitative analysis of (**c**) hNA-NeuN and (**d**) hNA-GFAP double-positive cells in the ipsilateral IBZ. Data are presented as mean values ± SD. Nuclei were counterstained with DAPI (*blue*). Scale bar, 20 μm.

Transdifferentiation of MSCs into cells of neural lineage has been reported [5-7]. To test the *in vivo *transdifferentiation of hUCB-MSCs administered intrathecally or intravenously, the neuronal marker NeuN and the astrocyte marker GFAP were evaluated at four weeks in the peri-infarct tissue of treated animals (Figure 3). hUCB-MSCs survived for at least four weeks and a subset of the grafted cells expressed NeuN and GFAP in the ipsilateral ischemic boundary zone in animals in which cells were administered intrathecally. Furthermore, a small subset of the grafted cells was immunopositive for CD73 and CD105, which are markers of MSCs for the state of non-differentiated cells (Additional file 2, Figure S2).

### Therapeutic effects of transplanted hUCB-MSCs

**Figure 4:**
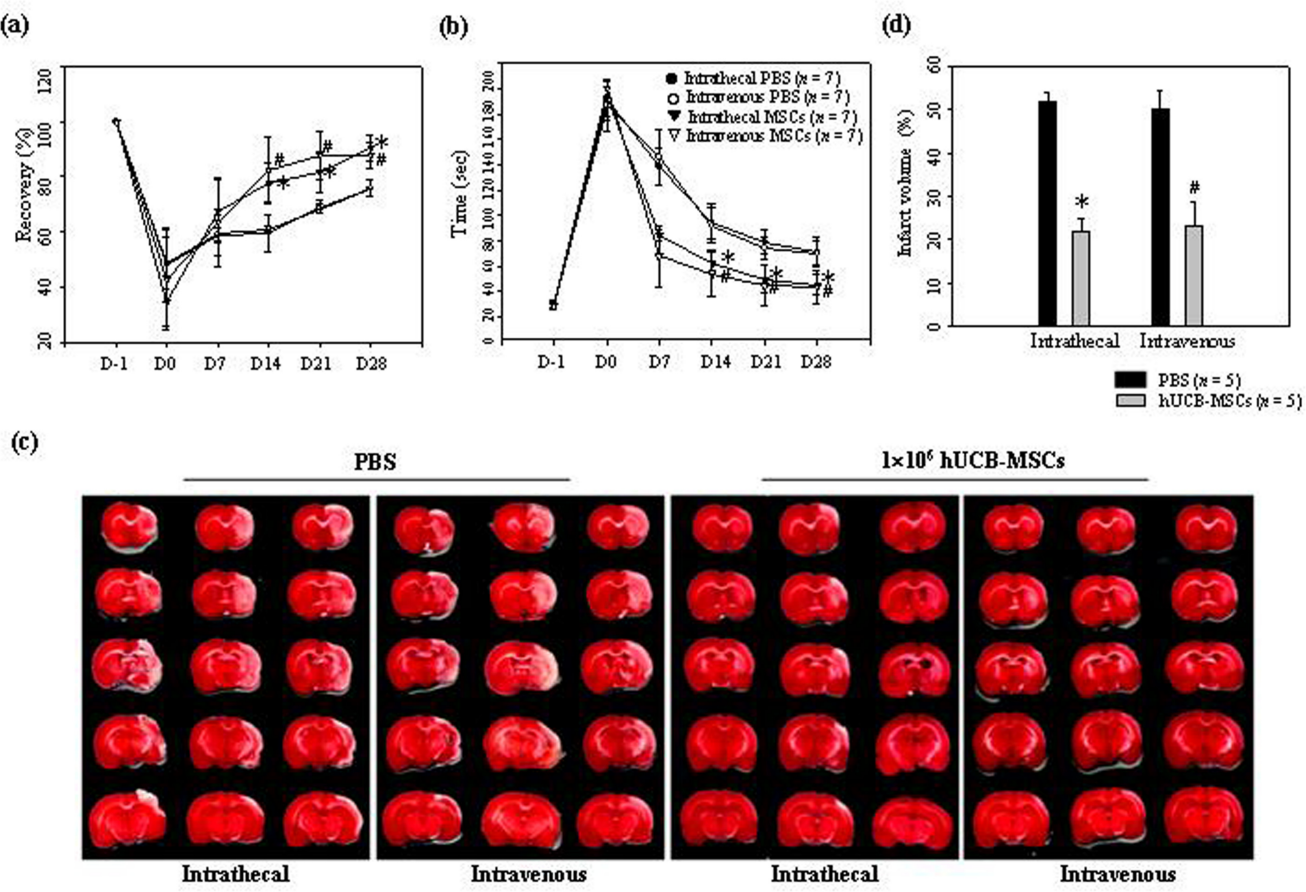
**Therapeutic effects of hUCB-MSC administration on recovery in the ischemic animal model**. Performance in the (**a**) Rotarod and (**b**) adhesive removal tests from 1 to 28 days after ischemia. The data were collected from seven animals per group and are presented as mean values ± SD. (**c**) Brain slices were stained with TTC at seven days after PBS or 1 × 10^6 ^hUCB-MSC administration to visualize lesions. (**d**) The data were collected from five animals per group and are presented as mean relative infarct volume ± SD. Statistically significant differences between the groups were determined by analysis of variance (*P < 0.05 compared with the PBS injected group intrathecally; #*P *< 0.05 compared with the PBS injected group intravenously).

We tested whether hUCB-MSCs administered intrathecally or intravenously enhanced neurological dysfunction. After treatment with 1 × 10^6 ^cells, motor function was analyzed using a Rotarod apparatus and an adhesive removal test in each group. One day after MCAO, but prior to intrathecal or intravenous administration of hUCB-MSCs, there was no difference in neurological functional assessment between the two ischemic groups. PBS-injected animals spontaneously recovered to a limited degree over 21 days. Animals that received hUCB-MSCs intrathecally or intravenously exhibited higher recoveries over 21 days than PBS treated animals and continued to recover for up to 28 days. At 28 days after injection, there were significant differences (*P *< 0.05) between hUCB-MSCs (90.35% ± 4.48%) and PBS (78.83% ± 3.22%) administered groups intrathecally in the Rotarod test and adhesive removal test scores (23.6 ± 10.57 vs. 49.77 ± 10.1, *P *< 0.05). There were also significant differences between hUCB-MSCs and PBS administered groups intravenously in the Rotarod test (87.76% ± 4.5% vs. 75.89% ± 0.3%, *P *< 0.05) and adhesive removal test scores (25.58 ± 7.6 vs. 51.65 ± 9.1, *P *< 0.05) (Figure 4a, b). However, there were no significant differences between the intrathecal and intravenous injection groups. Animals that received hUCB-MSCs showed good neurological recovery independently of the administration route, intrathecal or intravenous, without significant differences in neurological score.

The volume of ischemic lesions at seven days after the experimental procedure was evaluated using TTC staining. Administration of 1 × 10^6 ^hUCB-MSCs intrathecally (21.93% ± 2.85% vs. 51.68% ± 2.43%) or intravenously (23.22% ± 5.67% vs. 50.26% ± 4.18%) significantly reduced (*P *< 0.05) the infarction volume when compared with the PBS controls. There was no significant difference between the intravenous or intrathecal hUCB-MSCs treatment (Figure 4c).

**Figure 5:**
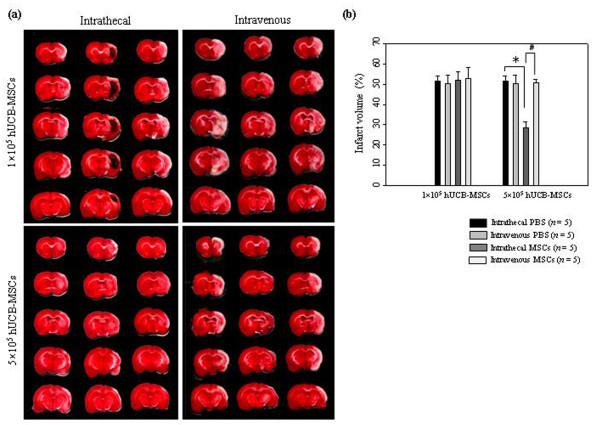
**Therapeutic effects of low-dose hUCB-MSC administration on infarction volume in the ischemic animal model**. (**a**) Brain slices were stained with TTC at seven days after 5 × 10^5 ^hUCB-MSC administration. The images show the lesion volume in hUCB-MSCs treated groups. (**b**) The data were collected from five animals per group and are presented as mean relative infarct volume ± SD. Statistically significant differences between the groups were determined by analysis of variance (*P < 0.05 compared with the PBS injected group intrathecally; #*P *< 0.05 compared with the hUCB-MSCs injected group intravenously).

Importantly, intrathecal administration of 5 × 10^5 ^hUCB-MSCs significantly the infarction volume when compared with the PBS controls (27.35% ± 3.17% vs. 51.68% ± 2.43%, *P *< 0.05). In addition, there were no significant differences in infarction volume between intrathecal injection of 5 × 10^5 ^and 1 × 10^6 ^hUCB-MSCs. However, intravenous administration of 5 × 10^5 ^hUCB-MSCs showed no effect on the infarction volume when compared with the PBS controls (50.52% ± 1.92% vs. 50.26% ± 4.18%). No effect on the infarction volume was seen with 1 × 10^5 ^hUCB-MSCs in either treatment group (intrathecal, 51.84% ± 4.15%; intravenous, 52.58% ± 5.8%) (Figure 5).

### Homing and survival of low-dose hUCB-MSCs in the ischemic brain

**Figure 6:**
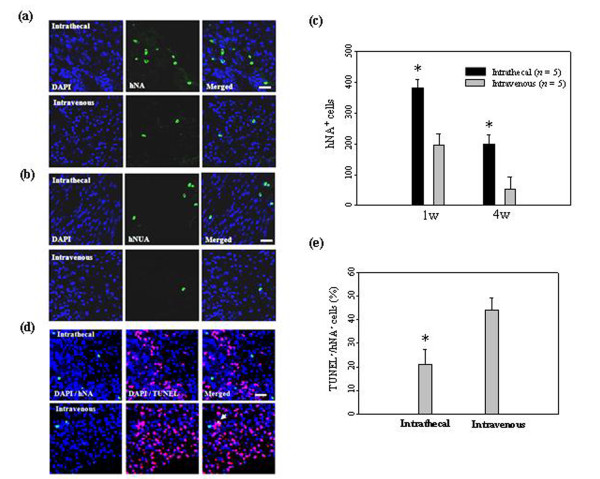
**Migration and survival of low-dose hUCB-MSCs in the ischemic brain**. (**a**) At 7 days and (**b**) 28 days after 5 × 10^5 ^hUCB-MSC administration, hUCB-MSCs were identified by the staining with human nuclei antibody (hNA, *green*) and the numbers of hNA-positive cells in the ischemic boundary zone (IBZ) of Ipsi hemisphere are illustrated (*n *= 5 per treatment group). (**c**) Data are presented as mean numbers of hNA-positive cells ± SD. Note that the numbers of hNA-positive cells were decreased in animals after intravenous administration compared with animals after intrathecal administration. Intrathecally treated groups showed significant differences from the intravenously treated groups in the IBZ. (**d**) At seven days after 5 × 10^5 ^hUCB-MSC administration, hUCB-MSCs undergoing apoptotic cell death were measured by TUNEL staining. The numbers of hNA-TUNEL double-positive cells in the ipsilateral IBZ are illustrated. (**e**) Quantitative analysis of hNA-TUNEL double-positive cells in the ipsilateral IBZ. Data from five animals are presented as mean values ± SD. There were significantly more hNA-TUNEL double-positive cells in animals after intravenous administration (analysis of variance; *P < 0.05). Nuclei were counterstained with DAPI (*blue*). Scale bar = 20 μm.

After administration of 5 × 10^5 ^hUCB-MSCs intrathecally or intravenously, migrated cells were observed at 7 and 28 days in the ischemic brain (Figure 6a-c). Many of the cells were found at seven days in the peri-infarct zone of the ischemic hemisphere in animals after intrathecal administration. However, a small number of cells were detected within the ischemic brain after intravenous administration.

To assess the survival of transplanted cells, a TUNEL assay was used to evaluate apoptosis of grafted cells in ischemic animals. One week after cell administration, 21% ± 6.2% of hNA-positive cells were stained for TUNEL (in the penumbra regions such as the ischemic boundary zone in animals administered hUCB-MSCs intrathecally (Figure 6d)). However, 44% ± 5.1% of hNA-positive cells were stained in animals in which they were administered intravenously (Figure 6e).

### *In vivo *differentiation of low-dose hUCB-MSCs in the ischemic brain

**Figure 7:**
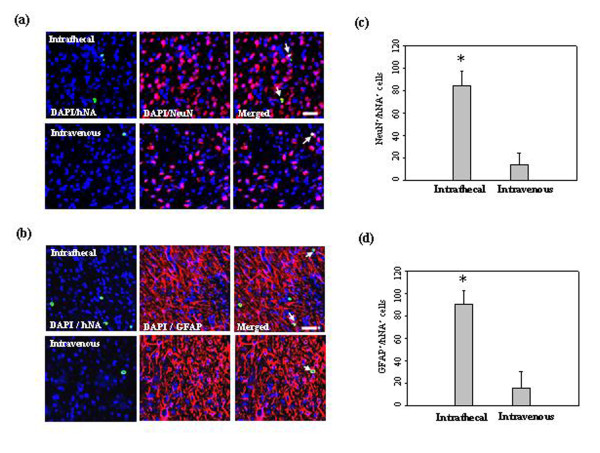
***In vivo *differentiation of low-dose hUCB-MSCs in the ischemic brain**. Confocal images of the cells at four weeks after 5 × 10^5 ^hUCB-MSC administration in the ischemic animal models. hUCB-MSCs were identified by the staining with human nuclei antibody (hNA, *green*). A subset of the grafted cells expressed (**a**) NeuN and (**b**) GFAP in the ipsilateral ischemic boundary zone. These markers were immunolabeled with red fluorescence. Quantitative analysis of (**c**) hNA-NeuN and (**d**) hNA-GFAP double-positive cells in the ipsilateral IBZ. Data are presented as mean values ± SD. There were significantly more double-positive cells in animals after intrathecal administration (analysis of variance; *P < 0.05). Nuclei were counterstained with DAPI (*blue*). Scale bar = 20 μm.

Immunolabeling showed that some of the grafted cells were positive for staining with the anti-Neun and GFAP antibodies in the ipsilateral ischemic boundary zone after intrathecal administration of hUCB-MSCs (Figure 7). Compared with intrathecal administration, a small subset of grafted cells expressed NeuN and GFAP.

## Discussion

Cell-based strategies are of particular interest in neurological conditions because mature brains have limited capacity for self-repair. MSCs have great potential as therapeutic agents for stroke treatment, because they are easily obtained and can be expanded rapidly *ex vivo *for transplantation [2,27]. MSCs transplanted into an ischemic region of the rat brain are capable of differentiation into neural cells and promote functional improvement [11,24,28]. Furthermore, MSCs can improve neurological dysfunctions in stroke patients [29]. However, it is often argued that stem cells might be used to replace lost neurons and restore functions [30].

hUCB-MSCs have proven to be more advantageous than bone marrow-derived MSCs in terms of cell procurement, storage, and transplantation [31]. Moreover, the number and differentiation ability of bone marrow-derived MSCs significantly decrease with age [32]. These characteristics make hUCB-MSCs potent candidates for the clinical application of allogenic MSC-based therapies.

The route of cell administration is a key point in stem cell transplantation. The need for development of effective cell delivery methods to enhance the therapeutic efficacy of stem cells is pressing because the safety and efficacy of cell therapy depend on the mode of cell administration. Several studies reported functional recovery in animal stroke models and in humans using different modes of delivery [33-35]. However, the optimal delivery route for cell transplantation after stroke is not yet well defined.

The present results demonstrate that administration of allogenic hUCB-MSCs intrathecally by LP is a valuable transplantation method for efficient cell delivery and therapy in a rat stroke model. Intravenous administration of 1 × 10^6 ^hUCB-MSCs is equally effective for improving neurological recovery and decreasing cerebral damage in ischemic stroke (Figure 4). A most important finding of the present study is that 5 × 10^5 ^hUCB-MSCs administered intrathecally are significantly effective for decreasing ischemic infarction volume, but not in the intravenous administration group (Figure 5). A relationship between cell dose and therapeutic effect has been identified by Chen and colleagues [12]. Rats intravenously infused with 3 × 10^6 ^MSCs after MCAO showed better neurological recovery than animals infused with 1 × 10^6 ^MSCs. Rats intravenously infused with 1 × 10^6 ^MSCs after MCAO showed improved neurological recovery, but rats administered 3 × 10^6 ^MSCs demonstrated better neurological recovery than animals infused with 1 × 10^6 ^MSCs. Although 1 or 3 × 10^6 ^cells in animal experiments are acceptable for therapeutic effect, extrapolation of these doses to humans may be difficult because of the large number of cells needed. This difficulty in converting the amount into a human dose will limit clinical trials. MSCs therapy for stroke patients has been performed using 1 × 10^8 ^cells [29,36]. A potential therapeutic effect at an acceptable cell dose is important in human therapy.

Homing is the process by which cells migrate to, and engraft in, the tissue in which they will exert functional effects [37,38]. Capacity for migration towards an injured region is an important characteristic of MSCs. When transplanted into the striatum or tail vein after MCAO, MSCs survived and migrated to the ischemic site, where they restored damaged neural cells in adult rodents [11,12,24]. The present study indicates that both administration routes were equally effective in neurological deficit recovery, but the intravenous administration did not produce MSC migration to the lesion zone (Figure 1). In addition, many more grafted cells survived in animals after intrathecal administration when compared with animals after intravenous administration (Figure 2). Our outcome suggests that it may not be necessary for the stem cells to successfully migrate and graft onto the lesion site to obtain good functional results.

Several factors are probably influential in achieving the benefits of MSCs in the ischemic brain, and a possible mechanism that could explain the improvement in functional recovery of models is believed to be associated with the differentiation of transplanted MSCs into a neural cell lineage. Numerous studies have reported that transplanted MSCs in animals with ischemic stroke expressed the neural cell lineage markers, such as the neuronal-specific protein NeuN, microtubule-associated protein 2 (MAP-2), and the astrocytic marker GFAP [11,24,28]. The neural differentiation capacity of MSCs *in vitro *and *in vivo *has been intensively explored; previous studies in our laboratory have also demonstrated that MSCs differentiate into neurons or glial cells *in vitro *under special experimental conditions [39,40]. In the present study, hUCB-MSCs delivered by LP grafted efficiently and differentiated into neurons and glial cells (Figures 3 and 7), supporting the hypothesis that transdifferentiation of transplanted MSCs is influential in achieving the benefits of MSCs in the ischemic brain.

On the basis of these results, both intrathecal and intravenous routes of administration of 1 × 10^6 ^cells have demonstrated similar effectiveness for promoting neurological recovery in ischemic stroke regardless of migration and grafting differences within the ischemic brain. However, intrathecal administration was significantly more effective for the 5 × 10^5 ^cell dose in reducing the ischemic damage. Our study indicates that intrathecal delivery of hUCB-MSCs by LP is an attractive and potentially successful method by which to treat stroke damage and may be a clinically feasible means of providing less invasive and repeatable transplantation therapy.

## Conclusions

Therapy with hUCB-MSCs is a potential treatment for ischemic stroke. Intrathecal administration of 1 × 10^6 ^hUCB-MSCs (high dose) and 5 × 10^5 ^cells (low dose) by LP demonstrated significant effects on recovery of ischemic damage. Therefore, intrathecal delivery of MSCs by LP may be a useful and feasible mode of administration for clinical treatment of brain injuries, such as stroke, or neurodegenerative disorders with MSCs.

## Abbreviations

CCA: common carotid artery; CSF: cerebrospinal fluid; ECA: exterior carotid artery; GFAP: glial acidic fibrillar protein; IBZ: ischemic boundary zone; LP: lumbar puncture; MCAO: middle cerebral artery occlusion; Neun: neuronal nuclei; PBS: phosphate buffered saline; ROI: region of interest; TTC: 2-3-5-triphenyltetrazolium; UCB-MSCs: umbilical cord blood-derived mesenchymal stem cells.

## Competing interests

The authors declare that they have no competing interests.

## Authors' contributions

CHJ and JAJ performed experiments and collected data. JWC and WIO prepared hUCB-MSCs. YH carried out the threshold image analysis of ischemic brain. SMK and CHR drafted the manuscript. JYL and SSJ contributed to conception and design of the study, interpretation of data and editing of the manuscript. All authors approved the final manuscript.

## Supplementary Material

Additional file 1 **Figure S1. Surface antigen characteristic of hUCB-MSCs**. Immunophenotyping of hUCB-MSCs. Cells at passage 6 were labeled with antibodies against the indicated antigens and then analyzed by flow cytometry. The results are representative of at least three independent experiments. Click here for file

Additional file 2 **Figure S2. Phenotype of transplanted hUCB-MSCs *in vivo***. Confocal images of the cells at four weeks after 1 × 10^6 ^hUCB-MSC administration in the ischemic animal models. hUCB-MSCs were identified by the staining with human nuclei antibody (hNA, *green*). A small subset of the grafted cells expressed (upper panel) CD73 and (bottom panel) CD105 in the ipsilateral ischemic boundary zone. These markers were immunolabeled with red fluorescence. Nuclei were counterstained with DAPI (*blue*). Scale bar: 20 μm. Click here for file
