# Supplementary figures and images for: Therapeutic effects of human umbilical cord blood-derived mesenchymal stem cells after intrathecal administration by lumbar puncture in a rat model of cerebral ischemia
Source: Stem Cell Res Ther. 2011 Sep 22;2(5):38. doi: 10.1186/scrt79 (PMC3308035; doi:10.1186/scrt79)

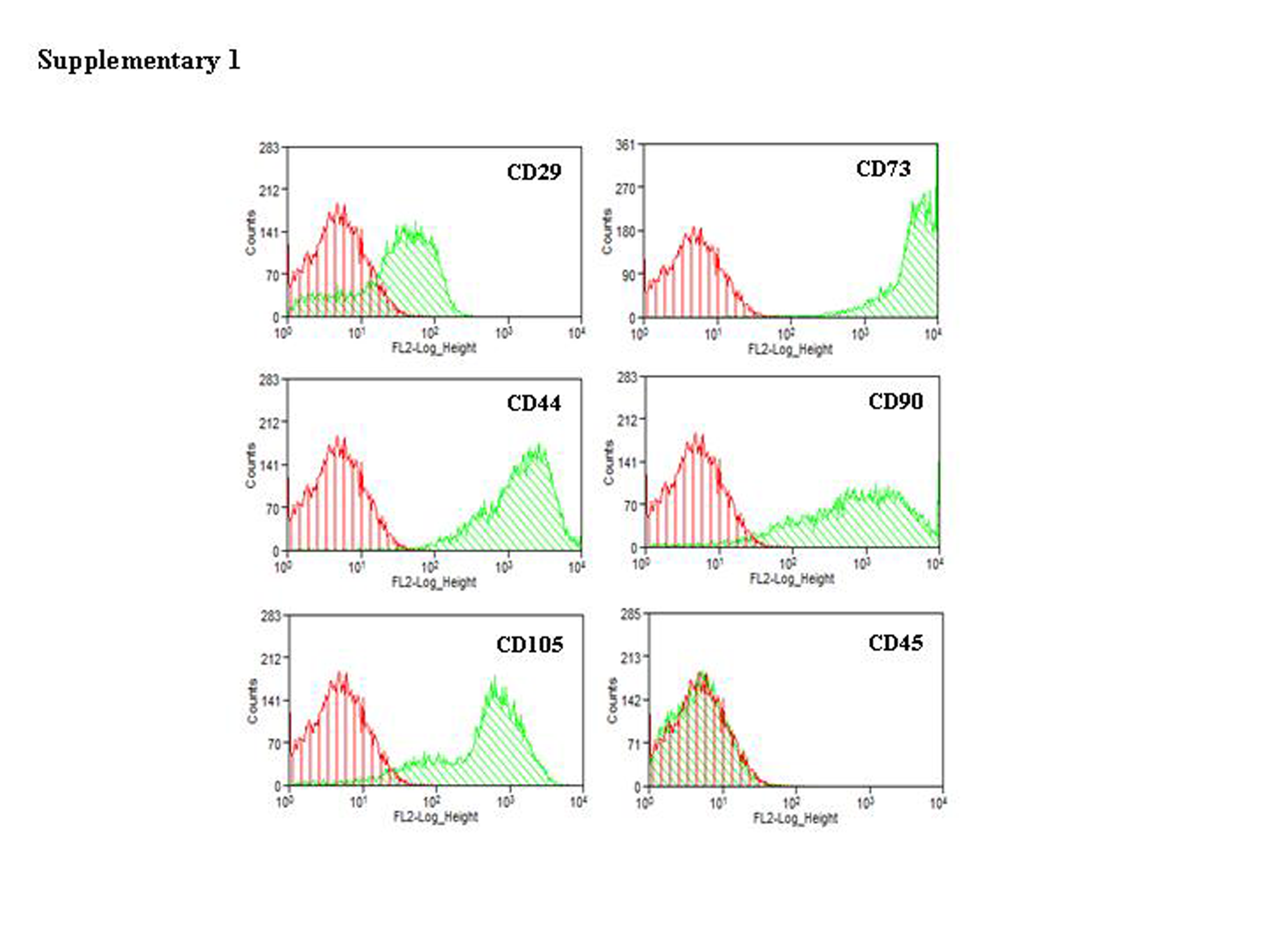

Supplement: Additional file 1 — Figure S1. Surface antigen characteristic of hUCB-MSCs. Immunophenotyping of hUCB-MSCs. Cells at passage 6 were labeled with antibodies against the indicated antigens and then analyzed by flow cytometry. The results are representative of at least three independent experiments. [file scrt79-S1.TIFF]

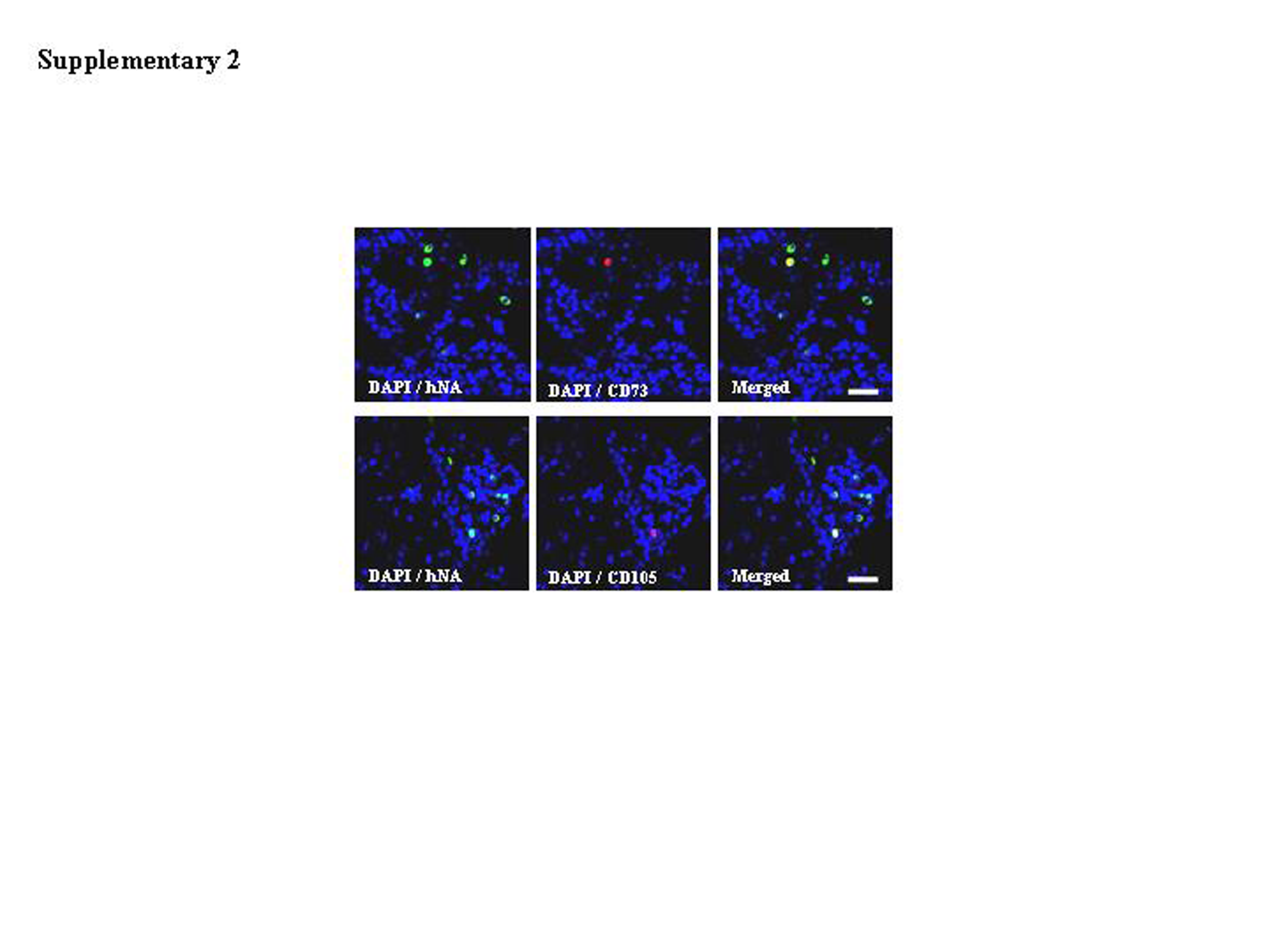

Supplement: Additional file 2 — Figure S2. Phenotype of transplanted hUCB-MSCs in vivo. Confocal images of the cells at four weeks after 1 × 10^6^ hUCB-MSC administration in the ischemic animal models. hUCB-MSCs were identified by the staining with human nuclei antibody (hNA, green). A small subset of the grafted cells expressed (upper panel) CD73 and (bottom panel) CD105 in the ipsilateral ischemic boundary zone. These markers were immunolabeled with red fluorescence. Nuclei were counterstained with DAPI (blue). Scale bar: 20 μm. [file scrt79-S2.TIFF]
